# Comparative Study on Vibration Fatigue Characteristics of Fuel Tank Material in Fuel and Air Media

**DOI:** 10.3390/ma13102372

**Published:** 2020-05-21

**Authors:** Long Yang, Bing Yang, Guangwu Yang, Sheng Dai, Xinhua Hua, Shangyuan Liu

**Affiliations:** State Key Laboratory of Traction Power, Southwest Jiaotong University, Chengdu 610031, China; yanglong6788@163.com (L.Y.); yb@swjtu.edu.cn (B.Y.); daisheng@my.swjtu.edu.cn (S.D.); 18628792614@163.com (X.H.); lsygzmail@163.com (S.L.)

**Keywords:** fuel tank material, virtual mass method, fuel medium, air medium, vibration fatigue test

## Abstract

To quantify the influence of the fuel medium on the fatigue performance of fuel tank materials, a comparative study was performed on the vibration fatigue characteristics of parent material specimens in fuel and air media. A fluid–solid coupling model was established based on the virtual mass method. Meanwhile, vibration fatigue tests of Q235BF specimens were performed in fuel and air media. The quantitative relation of the fatigue life of specimens in the air medium and that in the fuel medium was obtained. Fracture observation and energy spectrum analysis revealed the influence law of fuel on notches of specimens. The modal analysis of the finite element model proves that the stress of the specimens in the fuel medium is larger than that in the air medium. In this condition, they have shorter life. Finally, four approaches were used to calculate the fatigue life of the specimens to compare with the test life. The reasonable fatigue life prediction method was obtained in the case of fuel and air media.

## 1. Introduction

Fuel tanks are key components of the energy supply system for vehicles, such as diesel locomotives, automobiles, airplanes, and ships, and are under complex loads in operation. Owing to the vibration of the fuel inside the fuel tank, the fuel tank will be deformed under the action of the fuel load, which, in turn, will affect the size and distribution of the fuel load [1]. The fuel tank of a diesel locomotive is welded and fixed to the fuel tank hanger on the bottom beam and hangs on the bottom of the locomotive [2]. It has to withstand not only the impact and vibration generated by the track irregularity, but also the fluid–solid coupling effect caused by fuel vibration. The coupling between fuel and the tank is a highly nonlinear and stochastic process, presenting a very complex dynamic problem [3]. Under complicated loading conditions, stress is likely to be concentrated at the weld seam or other weak positions of the fuel tank and these parts will undergo fatigue cracking; hence, it is necessary to perform vibration fatigue analysis.

The commonly used material for diesel fuel tanks of a diesel locomotive is Q235BF. Owing to the large volume and mass of the diesel fuel tanks, studies on fatigue characteristics are mainly performed on the materials and scale models. For the fatigue test of fuel tank material, Wang et al. [4] conducted a three-point bending test on Q235 specimens and studied the fatigue characteristics of specimens with different-sized notches. The results showed that the fracture ductility varied with the notch size and was almost constant under elastic ultimate load. The bearing capacity of the structure was estimated using the relationship between the crack stress intensity factor and the fracture criterion. Han et al. [5] conducted low-cycle torsion and multi-axial fatigue tests on welded thin-walled Q235B specimens under different loading paths. The results showed that there was a significant non-proportional additional strengthening effect at a 90° out-of-phase loading path, and the authors proposed energy-based fatigue parameters that could better predict the fatigue life of Q235B steel structures under torsional and multi-axial loads. Pan [6] established a fatigue damage assessment model based on the fusion of magnetic-thermal characteristic data and verified the feasibility of the model via the axial tensile fatigue tests of Q235 specimens, accurately predicting the fatigue life of Q235 support rods. Gao [7] performed uniaxial tension and compression fatigue tests, torsional fatigue tests, and three-stage variable-amplitude loading fatigue tests on Q235 steel under different cyclic loading conditions and studied the correlation of fatigue damage to mechanical parameters such as specimen elongation, area reduction, tensile failure elongation, and ultimate tensile strength.

In terms of the influence of different media environments on material characteristics, Lynch [8] compared the scanning electron microscope (SEM) images of fatigue fracture of aluminum alloys in air, vacuum, and liquid environments, pointing out that more ductile cracks were formed in air and severe fracture passivation occurred in vacuum. There was no striation, and the liquid environment was more likely to cause the formation of brittle cracks. Bogdański [9] studied the comparison of quasi-static and hydrodynamic modelling of liquid-solid interaction in 3D squat-type cracks. The hydrodynamic interaction was modeled by coupling the 3D finite element model of a squat-type crack and the theoretical squeeze fluid film model of liquid contained in the crack interior. The squeeze fluid film in the central plane perpendicular to the crack front was modeled on the basis of the one-dimensional Reynolds equation derived for compressible fluid with pressure dependent viscosity. An opinion about the ability of “sucking in” a liquid (for instance oil or water) into the crack interior during single cycle of opening was verified. Bogdański [10] used the iterative numerical procedure to estimate the 3D crack front loading enhancements due to the action of the “liquid entrapment mechanism” (LEM). The 3D shallow angle, semi-elliptical, surface breaking crack inclined at 20° to the horizontal was FE modeled and used for analysis. The histories of modes I, II, and III stress intensity factors (SIFs) and corresponding fatigue growth rates were determined for the selected points of the crack front. The obtained loading enhancements were compared with those estimated earlier by the authors and others on the basis of the 2D model of LEM. Sun et al. [11] used a simplified submarine-stern model as the research object and established the relationship between the excited vibration and radiated noise of the model in air and water media.

The problems commonly encountered in fluid–solid coupling mainly include the dam–water coupling problem in civil engineering, the problem of fluid-induced pipeline vibration, the ship–water coupling problem, and the oscillation problem of liquid storage container [12]. The classical methods to solve the fluid–solid coupling problem consist of analytical methods and semi-analytical methods. With the advancement of computational techniques, numerical methods such as finite element methods and boundary element methods have been widely used [1]. There are many finite element methods for fluid–solid coupling problems, including the fluid–solid coupling method, hydroelastic fluid unit method, virtual mass method [13,14,15,16], fluid element method [17], central mass method [15], and acoustic–solid coupling method [16]. In particular, the virtual mass method simplifies the combined element equation of liquid–solid coupling to a structural finite element equation containing an attached mass matrix of the liquid, which has the advantages of high solution precision and small calculation load. Liu [18] studied the relationship between the maximum tank natural frequencies of dry modes and wet modes for different baffle positions using the virtual mass method, and the results provided a numerical basis for designing fuel tank structure and improving performance. Mantecón et al. [19] modeled fluids using ANSYS CFX software, modeled nuclear fuel plates using software ANSYS Mechanical, and then performed fluid–solid coupling analysis, proposing a numerical method for fluid–solid coupling analysis of nuclear fuel plates under axial flow conditions. Costarelli [20] proposed a Navier–Stokes solver based on Cartesian structured finite volume discretization with embedded bodies and adopted an explicit partitioned strategy to realize fluid–solid coupling.

The above studies mainly focused on the conventional fatigue characteristics of materials, the effects of gas and liquid media, fluid–solid coupling analysis, and simulation methods. However, there have been no studies on the vibration fatigue characteristics of fuel tank materials in fuel and air media.

**Figure 1:**
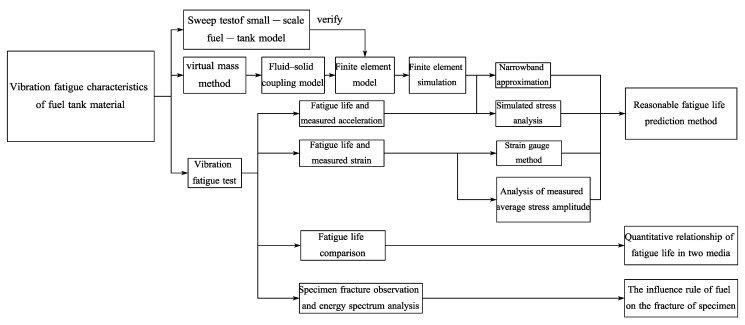
Flowchart of research method.

In this study, a fluid–solid coupling model of a small-scale fuel tank for a certain type of diesel locomotive was established using the virtual mass method. The validity of the model was verified using a frequency sweep test, and then comparative tests were performed on the vibration fatigue of specimens made of fuel tank materials under multi-stage loading in fuel and air media. Based on this, a fluid–solid coupling model was established for finite element simulation, and four fatigue calculation methods were compared, revealing the fatigue life calculation methods suitable for different media. The findings would provide a reference for predicting the fatigue life of fuel tank materials. The flowchart of the research method for comparing the vibration fatigue performance of fuel tank materials in the fuel and the air medium is shown in Figure 1.

## 2. Fluid–Solid Coupling Model and Verification

### 2.1. Fluid–Solid Coupling Model

When solving fluid–solid coupling problems, the virtual mass method has the advantages of high solution precision and small calculation load. In this study, the virtual mass method was used to study the effects of mutual coupling between fuel and specimens in fuel tanks. With the consumption of fuel, the total mass of the fuel tank of a diesel locomotive is continuously reduced, and the natural frequency constantly changes. If the frequency of the fuel tank is close to the excitation frequency of the engine and the motor, resonance will occur.

The calculation of natural frequency and vibration mode is a typical eigenvalue problem. The eigenvalue corresponds to the natural frequency, and the eigenvector corresponds to the vibration mode. The typical forced vibration equation with N degrees of freedom is [1]:

(1) $$\left[M\right]\left\{\ddot{u}\right\}+\left[C\right]\left\{\dot{u}\right\}+\left[K\right]\left\{u\right\}=\left\{F\right\}$$

In Equation (1), [*M*] is a mass matrix, [*C*] is a damping matrix, [*K*] is a stiffness matrix, *F* is the external load, and {*u*} is a displacement vector.

If the equation ignores the effects of damping and external load, assuming that the deformation of the fuel tank is within the linear elastic range of the material, the equation becomes [21]:

(2) $$\left[M\right]\left\{\ddot{u}\right\}+\left[K\right]\left\{u\right\}=0$$

A simple harmonic excitation as shown in Equation (3) is applied to the specimen in the fuel tank:

(3) {u}={ϕ}sinωt

In Equation (3), *ϕ* is an amplitude vector of the simple harmonic excitation, *ω* is the frequency, and t is time.

Under the action of simple harmonic excitation, the specimens and fuel in the fuel tank are subjected to acceleration, and the specimens in the fuel medium are more accelerated than the specimens in the air medium:

(4) $$F_{A}^{\prime }=\left(m_{A}+m_{A}^{\prime }\right)a>F_{A}=m_{A}a$$

In Equation (4), $m_{A}^{\prime }$ is the mass of the fuel attached to the specimens in the fuel tank.

The core of the virtual mass method is to achieve the effect of an incompressible fluid on the structure by introducing an attached mass and stiffness matrix in Equation (2). The finite element calculation equation for the vibration mode of the structure in the fuel medium is [14,15,16,18]:

(5) $$\left[M+M_{A}\right]\cdot \left\{\ddot{u}\right\}+\left[K+K_{A}\right]\cdot \left\{u\right\}=0$$

In Equation (5), *M_A_* is the attached mass matrix produced by the fluid to the structure, with $\ddot{u}$ and *u* as the acceleration and displacement vectors, respectively.

Substitution of Equation (3) into Equation (5) yields:

(6) $$-\omega^{2}\left[M+M_{A}\right]\left\{\phi \right\}\sin\omega t+\left[K+K_{A}\right]\cdot \left\{\phi \right\}\sin\omega t=0$$

As Equation (6) holds at any time, one obtains:

(7) $$\left[K+K_{A}\right]-\omega^{2}\left[M+M_{A}\right]\left\{\phi \right\}=0$$

The prerequisite for Equation (7) to have a non-zero solution is that the characteristic determinant is zero:

(8) $$\det\left[K+K_{A}\right]-\omega^{2}\left[M+M_{A}\right]\left\{\phi \right\}=0$$

A series of eigenvalues *ω*_i_ can be obtained from Equation (8), each corresponding to an eigenvector $\left\{\phi_{i}\right\}$ satisfying Equation (7). Each eigenvalue and the corresponding eigenvector satisfy Equation (9), wherein the eigenvalue corresponds to a natural frequency, and the eigenvector corresponds to a vibration mode [22]:

(9) $$\left[K+K_{A}\right]-\omega_{i}{}^{2}\left[M+M_{A}\right]\left\{\phi_{i}\right\}=0$$

As shown by Equation (5), on the one hand, the attached mass matrix changes with the state of the fluid, and the vibration of the structure is a state function of the fluid flow; on the other hand, the vibration of the structure affects the fluid flow state through boundary deformation, and the fluid and fuel tank form a closed fluid–solid coupling system. As the stiffness *K*_A_ imposed by the fluid on the fuel tank is small relative to the stiffness of the fuel tank itself, it can be ignored. Consequently, the attached mass matrix *M_A_* is the main research object.

The following three assumptions are made to solve the equation of attached mass matrix *M_A_* [15,18]:

1. The liquid is an isotropic, incompressible, non-viscous liquid;
2. The influence of gravity on the surface of the structure can be ignored;
3. The motion speed of the structure is very low.

According to the continuity, motion, and energy equations of fluid mechanics, assuming that $\left\{\sigma_{j}\right\}$ and $\left\{{\dot{\sigma }}_{j}\right\}$ are, respectively, the fluid velocity and fluid acceleration calculated via the boundary element method at point r_j_ (a virtual source) on the boundary of the fluid domain, the flow velocity $\dot{u}$ and pressure *p_i_* at another position *r_i_* on the boundary of the fluid are expressed, respectively, by Equations (10) and (11) [23]:

(10) $$\dot{u}=\sum_{j}\underset{A_{j}}{\int }\frac{\sigma_{j}e_{ij}}{{\left|r_{i}-r_{j}\right|}^{2}}dA_{j}$$

(11) $$p_{i}=\sum_{j}\underset{A_{j}}{\int }\frac{\rho \dot{\sigma_{j}}e_{ij}}{\left|r_{i}-r_{j}\right|}dA_{j}$$

In Equations (10) and (11), ${\dot{u}}_{i}$ is the velocity vector at any node; *A_j_* is the area of a microelement on the surface of the structure; $\sigma_{j}$ is the flow velocity vector at node j; e_ij_ is the unit vector from node j to node i; *p_i_* is the pressure on an arbitrary surface *A_j_*; ρ is the fluid density.

Integration of Equations (10) and (11) yields Equations (12) and (13) [24]:

(12) $$\left\{\dot{u}\right\}=\left[\chi \right]\cdot \left[\sigma \right]$$

(13) $$\left\{F\right\}=\left[Ʌ\right]\cdot \left[\dot{\sigma }\right]$$

where [χ] and [Ʌ] are integral coefficient matrices; *F* is the force exerted by the fluid on the structure.

According to Newton’s second law, Equations (12) and (13) can be converted into Equation (14):

(14) $$\left\{F\right\}=\left[Ʌ\right]\cdot {\left[\chi \right]}^{-1}\left\{\ddot{u}\right\}=\left[M_{A}\right]\left\{\ddot{u}\right\}$$

namely:

(15) $$\left[M_{A}\right]=\left[Ʌ\right]\cdot {\left[\chi \right]}^{-1}$$

According to Equation (15), the attached mass matrix *M_A_* can be obtained, which is substituted in Equation (5) to solve the equation for eigenvalues and eigenvectors, which are the natural frequencies and vibration modes of the fluid–solid coupling system, respectively. 

### 2.2. Model Validation

Frequency sweep tests were performed on a small-scale fuel-tank model of a diesel locomotive with different fuel levels. The test results were compared with the simulation results obtained based on the virtual mass method to verify the validity of the fluid–solid coupling model.

**Figure 2:**
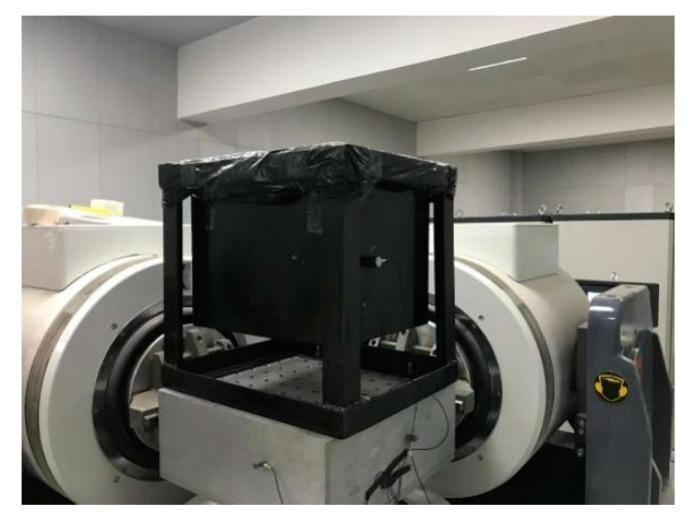
Schematic diagram of the test platform and model installation.

The small-scale fuel-tank model was installed on a multi-axial synchronous vibration platform for a sinusoidal frequency sweep test. The test platform and model installation are shown in Figure 2. 

Acceleration sensors were arranged on the two side plates and the bottom plate of the model. The lower and upper frequency limits of the sweep test were set to 5 Hz and 160 Hz in the vibration control system, respectively, with the sweep rate set to 1 oct/min.

The small-scale fuel-tank model was subjected to a sinusoidal frequency sweep test at 0/4, 1/4, 2/4, 3/4, and 4/4 of the full fuel level, which provided the first-order resonance frequency at each fuel level. When using the virtual mass method to simulate fluid–solid coupling of the small-scale fuel-tank model, the first step was to establish a local coordinate system at the bottom of the tank, and the second step was to define the fluid characteristics and the characteristics of fluid–solid coupling elements by modifying the MFLUID and ELIST control cards in the Msc Nastran 2010 software. The MFLUID and ELIST control cards can set the numbers of the continuous wetted elements, attribute and depth of the fluid. For example, ZFS defines the depth of the fluid, RHO defines the density of a fluid, E1 represents the number of the initial wetted elements and E3 represents the number of the terminal wetted elements. The parameters of the MFLUID and ELIST control cards are shown in Table 1. 

**Figure 3:**
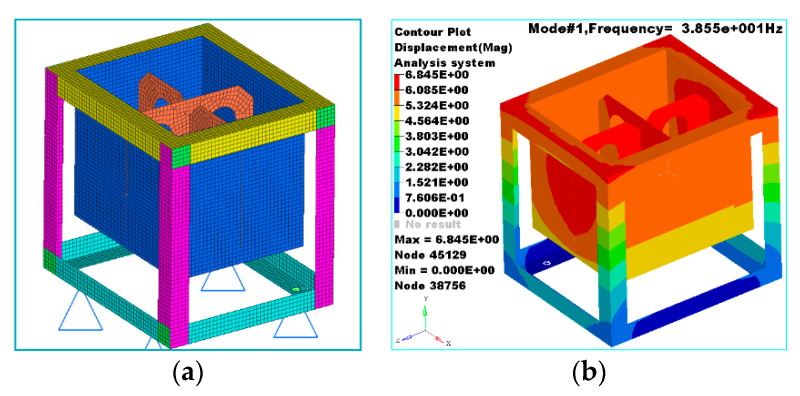
(**a**) Finite element model of the fuel-tank structure and (**b**) simulation results at 1/4 of full fuel level.

The small-scale fuel-tank finite element model and its modes at 1/4 of the full fuel level are shown in Figure 3a,b.

The results of the frequency sweep test of the small-scale fuel-tank model and the modal simulation results based on the virtual mass method are shown in Table 2. 

The results show that, with the increase in fuel height, the first-order frequency gradually decreased, which was attributed to the fact that the fluid mass was treated as the attached mass *M_A_* and absorbed in the mass matrix in the calculation. As shown by Equation (8), with the increase in *M_A_*, the eigenvalue *ω*_i_ decreased gradually, that is, the natural frequency of the system gradually decreased. The test and simulation results are consistent with the theoretical solution of the fluid–solid coupling model.

The simulation results of the fluid–solid coupling model based on the virtual mass method are very close to those of the frequency sweep test, showing a maximum relative deviation of only 5.73%. This indicates that it is feasible to conduct fluid–solid coupling simulation based on the virtual mass method, thereby verifying the accuracy of the model. The virtual mass method greatly reduces the difficulty of modeling because it does not need to establish fluid grid cells, thereby making it more suitable for the fluid–solid coupling simulation of large fuel tanks, such as those of diesel locomotives, airplanes, and ships.

## 3. Vibration Fatigue Test

### 3.1. Test Process

**Figure 4:**
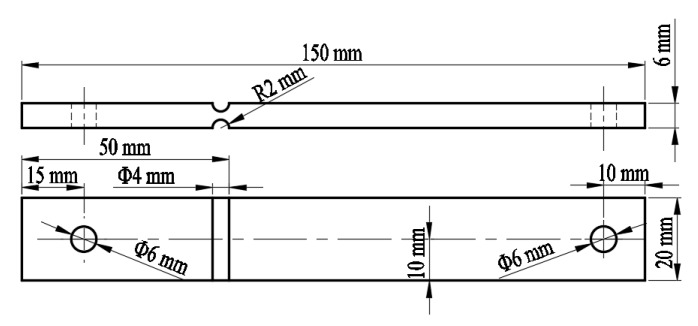
Schematic diagram of the specimen structure.

During the operation of the diesel locomotive, the fuel tank has to withstand not only the impact and vibration generated by the external load, but also the fluid-solid coupling effect caused by fuel vibration. Therefore, the multiaxial stress state occurred at the bottom and sides of the fuel tank. To study the influence of fuel and air media on the fatigue behavior and vibration fatigue characteristics of a fuel tank, parent material specimens were designed for the fuel tank of a type of diesel locomotive and subjected to vibration fatigue tests. The support and the mounting base were rigidly welded to ensure that the specimens would not be loose and the acceleration remained constant. In order to speed up the test and magnify the influence of fuel on fuel tank materials, the specimens were installed as cantilever beam structure to carry out the fully-reversed uniaxial bending tests. The geometry of the specimens is shown in Figure 4. 

The circular hole (Φ6 mm diameter) on the left side is used for assembly, and the circular hole (Φ6 mm diameter) on the right side is used to install the counterweight block. The specimen material was Q235BF. The chemical composition and physical and mechanical properties of Q235BF specimens are shown in Table 3.

**Figure 5:**
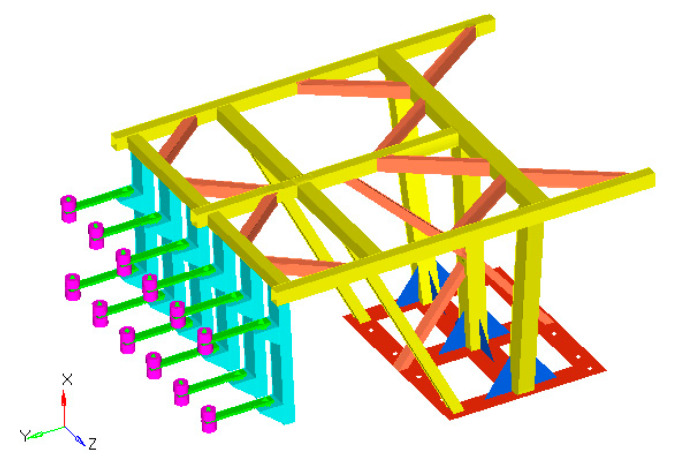
Finite element model.

**Figure 6:**
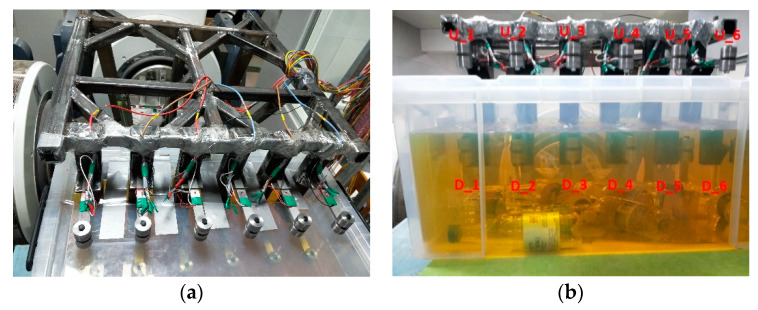
(**a**) Specimen support setup and (**b**) specimen numbering.

The mounting support is shown in Figure 5, and the connection diagram between the specimen and the vibration platform is shown in Figure 6a,b.

A total of 12 specimens were tested, six of which were in the air medium and six in the fuel medium. The acceleration was applied to rigid support in vertical direction (x-direction) through the surface of the multi-axial vibration platform. Then the acceleration was applied to the specimens through the support. The acceleration sensor was attached at the root of the specimens to feed back the acceleration information so that the acceleration could be kept constant. To make the input frequencies of the vibration platform different from the natural frequencies of the specimens and to determine the range of the input acceleration, simulation was first performed to identify structural modes and static strength.

The modes and static strength identified via modal simulation are shown in Table 4. 

The results show that the first-order resonance frequency of the structure was 29.22 Hz. To avoid resonance, the input frequency of the vibration platform should avoid selecting 29.22 Hz. From the S–N curves [25,26] of Q235BF-grade notched specimens, the corresponding stress amplitude at the life of 10^6^ cycles was 180 MPa. When the acceleration was 10.88 g, the first principal stress at the specimen notch was 179.9 MPa. If the test input frequency was 20 Hz, the test duration was expected to be approximately 13.89 h. In order to control the test duration reasonably and avoid resonance, the input frequency of the vibration platform was selected to be 20 Hz.

Most of the traditional multi-stage loading tests use the second- and third-stage blocks in the order of high, low, and high loading or in the order of low, high, and low loading [27]. Chu [28] analyzed the cumulative strengthening effect of the loading sequence on fatigue strength by conducting three-stage loading fatigue tests with different loading sequences, determining that the cumulative strengthening effect of training loading on fatigue strength was the highest when loading in the order of low to high magnitudes, and that the cumulative strengthening effect was similar to the additive strengthening effect of three stages of independent loading. To maximize the cumulative strengthening effect of exercise load on the fatigue strength and make the loading contain the main vibration state of the fuel tank, the input acceleration of the vibration platform was set to three-stage constant-amplitude sinusoidal excitation (5.18 g, 8.55 g, and 13.45 g), with the load applied in the order of small to large magnitudes.

The specimens in the air medium were attached with a resistance strain gauge at a position 8 mm from the left side of the notch and an acceleration sensor was installed to the mounting base of specimen support. The measured strain could be used to calculate the stress at the notch. The strain gauge can be affected by the fuel, and cannot measure stress accurately. Thus, the stress values of the specimens in the fuel medium were obtained by finite element simulation. The specimens in the air medium were named U_1–U_6 from left to right, and the specimens in the fuel medium were named D_1–D_6 from left to right. To facilitate the observation of the real-time state of the specimens during the test, a transparent plastic fuel tank was used to hold the fuel, and the specimens in the fuel medium were placed 60 mm below the fuel level.

### 3.2. Test Results

First, the test was performed for 7 h under a load of 5.18 g. The test results, as shown in Table 5, indicated that there was no specimen fracture at this stage.

Subsequently, the test was continued for 72.25 h under a loading of 8.55 g. The test results, as shown in Table 6, indicate that the number of specimens fractured in the fuel medium was higher than that in the air medium, with the former showing a shorter life. 

Finally, the test was continued under a loading of 13.45 g until the remaining specimens were all fractured. The test results, as shown in Table 7, indicate that, except for the sixth group, the other five groups of specimens showed a significantly longer life in the air medium than in the fuel medium.

Based on the results in Table 4, the S–N curves of Q235BF-grade notched specimens [25,26] and the Miner’s cumulation rule (*N* = *N*_i_/*N*_fI_), the test life at accelerations of 5.18 g and 13.45 g was converted to that at an acceleration of 8.55 g (Table 8).

## 4. Fracture Observation and Energy Spectrum Analysis

### 4.1. Fracture Observation

**Figure 7:**
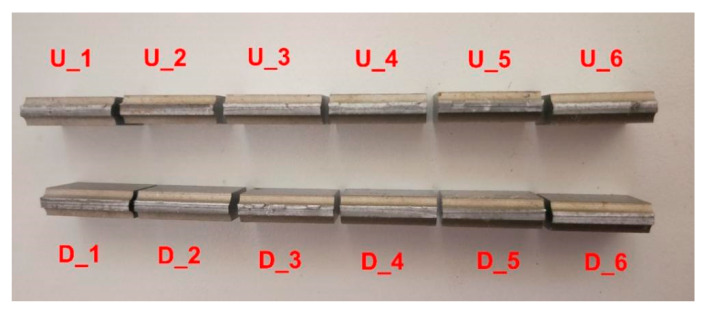
Macroscopic fracture morphology of all specimens.

In the vibration fatigue tests, as the input loading was a constant-amplitude sinusoidal acceleration, the specimens in the air medium were subjected to pure bending loading under the action of the counterweight block, and the specimens in the fuel medium were also mainly subjected to bending loading under the composite action of the counterweight block and fuel pressure. Consequently, the macroscopic fracture morphology of the specimens was the same. It was observed that the fatigue fracture occurred in a short time after the crack source was generated in the test. Thus, the fatigue life is mainly controlled by the crack source. As shown in Figure 7, the specimens were all fractured at the smallest section of the notch, and the fracture planes were perpendicular to the specimen axial direction.

**Figure 8:**
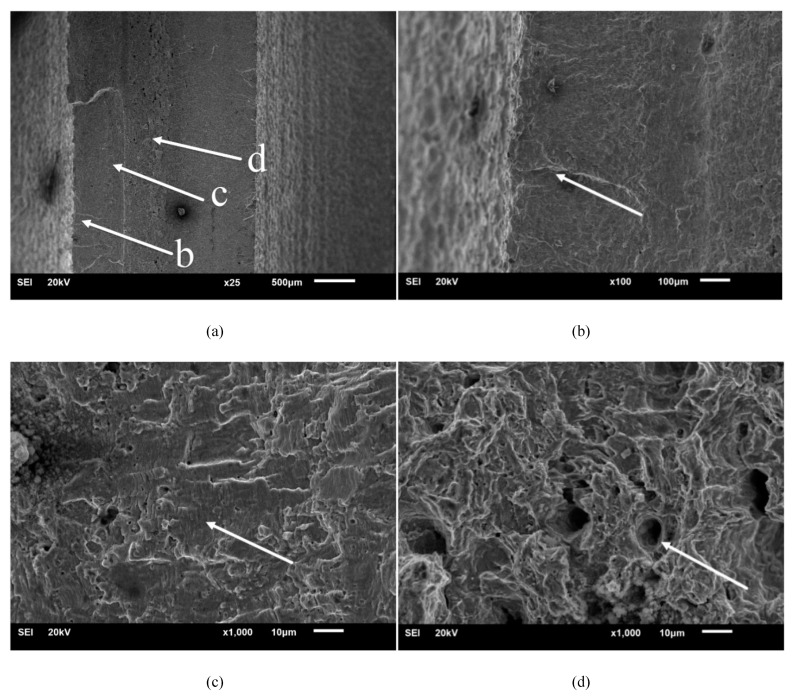
Fracture morphology of the U_1 specimen. (**a**) Macroscopic fracture, (**b**) crack source zone, (**c**) crack propagation zone, and (**d**) instantaneous fracture zone.

**Figure 9:**
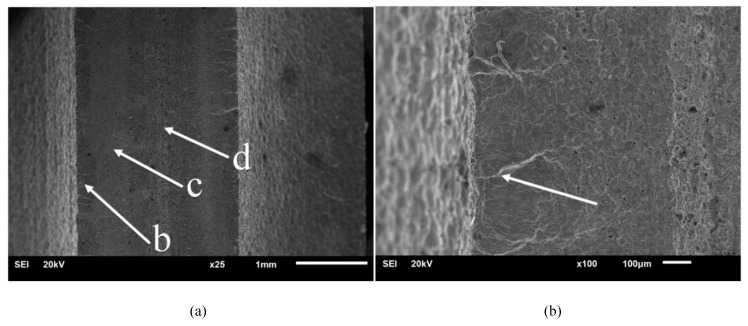

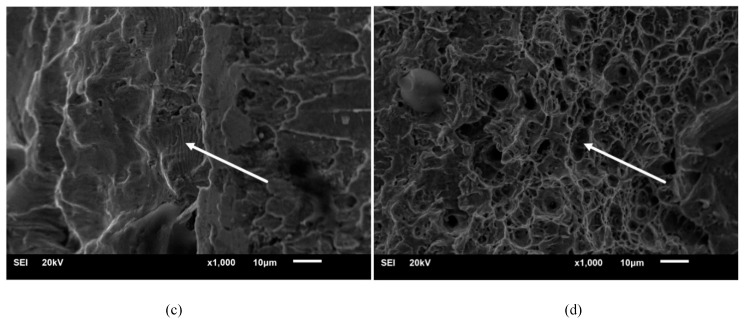
Fracture morphology of the D_1 specimen. (**a**) Macroscopic fracture, (**b**) crack source zone, (**c**) crack propagation zone and (**d**) instantaneous fracture zone.

To analyze the expansion pattern of fatigue cracking of the specimens and their initial defects further, the specimen fracture was observed under an SEM. Figure 8a–d and Figure 9a–d present the SEM diagrams of fracture morphology in the air and fuel media for the typical specimens U_1 and D_1, respectively. Figure 8a and Figure 9a,b represents the crack source zone, c represents the crack propagation zone, and d represents the instantaneous fracture zone.

**Figure 10:**
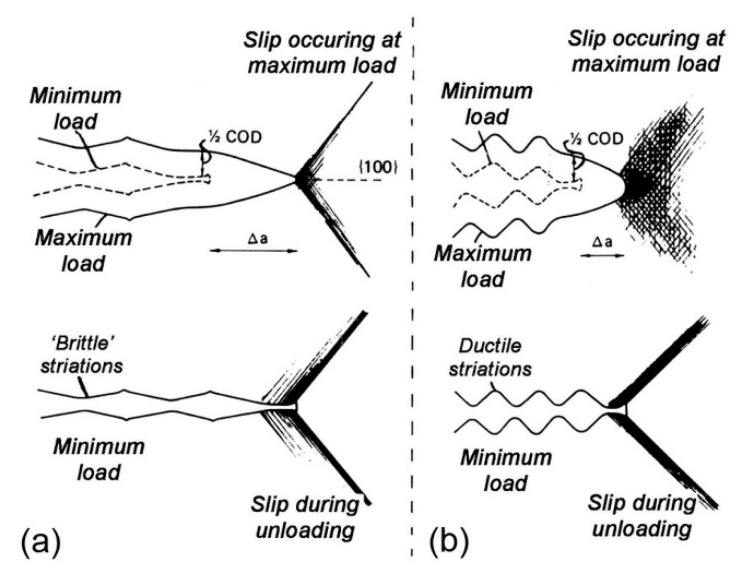
Crack propagation mechanism in a liquid environment [8]. (**a**) Liquid-metal environment producing brittle striations; (**b**) inert environments producing ductile striations.

A comparison between Figure 8a–d and Figure 9a–d reveals that, as the specimens were mainly subjected to bending loading in the air and fuel media, they had the same macroscopic fracture morphology, which was characterized by a typical failure of multi-crack source, with the crack sources concentrated on the left and right sides and expanding in a radial manner. Most of the instantaneous fracture zone was located in the center of the fracture, and the crack propagation zone was located between the crack source zone and the instantaneous fracture zone. However, the crack propagation zone of the specimen in the fuel medium was apparently more scratched and worn with fewer fatigue striations than that in the air medium, and the fatigue fringe is less. This was attributed to the fact that the compressive stress of the fuel in the process of crack tip opening and closing increased the stress intensity factor, which aggravated plastic sharpening, resulting in a relatively high crack propagation rate, fewer fatigue striations, and severe fracture wear. In this regard, Lynch [8] pointed out that, compared with an air environment, specimens in a liquid environment show greater crack propagation increments, lesser passivation, fewer fatigue striations, and smaller plastic zones with a higher possibility to generate split cracks. The mechanism of crack propagation in a liquid environment is shown in Figure 10 [8].

In addition, the dimples of the instantaneous fracture zone of the specimens in the fuel medium were significantly shallower and less irregular than those in the air medium. This is because, when the material ductility is high, the size of micro-dimples is large and deep [29].

**Figure 11:**
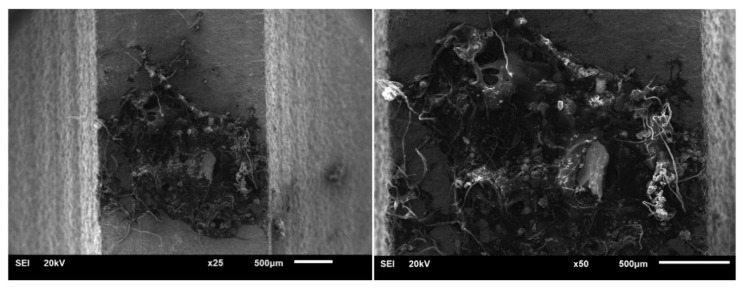
SEM diagrams of the dark gray zone of the U_6 specimen under 2five-fold and 50-fold magnification.

Notably, as mentioned in Section 2.2, the sixth group of specimens had a shorter life in the air medium than in the fuel medium. Fracture observation and energy spectrum analysis revealed that there were obvious foreign bodies on the fracture of the U_6 (Figure 11) specimen and the results of the energy spectrum analysis revealed that the chemical composition of the foreign bodies is quite different from that of the base mental. 

Thus, the foreign bodies should be manufacturing defects. Therefore, the test results of this group were excluded from further analysis. Based on the test data of the first five groups of specimens in Table 8, the average ratio of converted life in the air medium to converted life in the fuel medium was calculated to be 10.03.

### 4.2. Energy Spectrum Analysis

**Figure 12:**
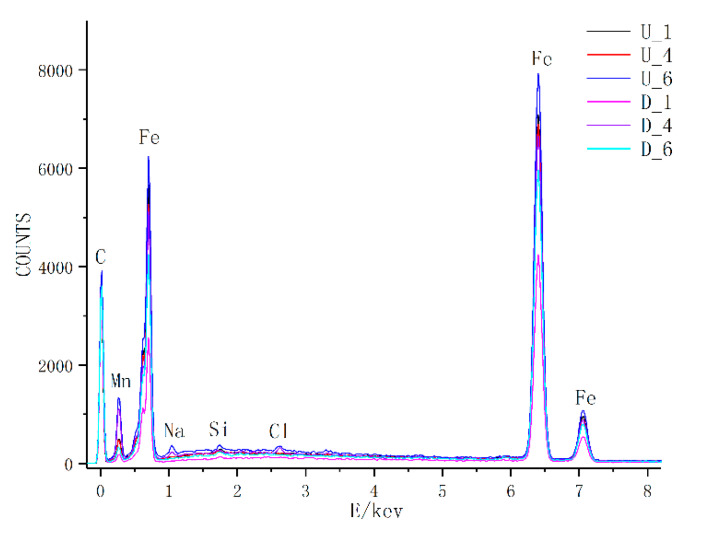
Results of energy spectrum analysis at a set of fixed measurement points.

To determine whether there was fuel corrosion and whether the crack source zone underwent chemical reactions with the fuel in the specimen fatigue process in the fuel medium, energy spectrum analysis was performed on the crack source zones of typical specimens. For each specimen, four measurement points were selected, and the results of energy spectrum analysis are shown in Figure 12.

As shown in Figure 12 (U_1–U_6 in the label are the specimens in the air medium, D_1–D_6 in the label are the specimens in the fuel medium), there was no significant difference between the air and fuel media in the energy spectrum analysis results of crack source zone, and the two media showed similar elemental composition and distribution in the specimens. This indicates that the crack source zone of the specimens in the fuel medium did not undergo chemical reactions with the fuel. The formation and propagation of cracks did not result from fuel erosion but resulted from the superposition of the bending stress at the crack tip with the compressive stress of the fuel.

## 5. Modal and Fatigue Life Comparison

### 5.1. Modal Comparison

To make the simulation test accurate, a fluid–solid coupling model of the specimen was established in a 1:1 ratio using the virtual mass method. The specimen, support, and fuel tank were discretized by the body element and the shell element, respectively. The fuel was simulated by establishing a local coordinate system on the bottom of the fuel tank and defining the MFLUID and ELIST control cards. The wetted elements were renumbered to become continuous. The normal directions of the wetted elements were adjusted to point to the fuel, and the serial numbers of the wetted elements were defined in the ELIST control card.

**Figure 13:**
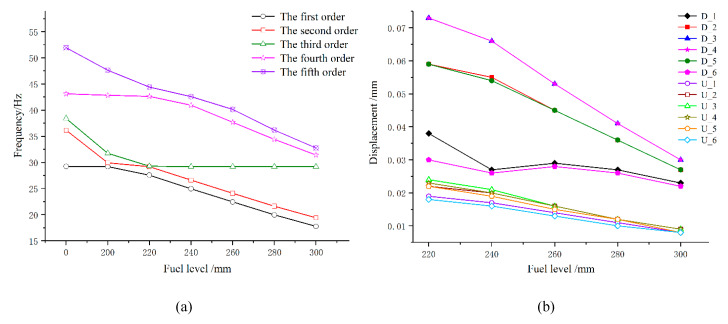
Modal frequency and displacement of the structure. (**a**) Modal frequency and (**b**) modal displacement.

In the tests, the distance from the upper surface of the specimens in the fuel medium to the bottom of the fuel tank was 200 mm; hence, 200 mm was used as the baseline level for fuel filling. The wet modes of the structure at the filled fuel level of 200–300 mm were simulated via the virtual mass method, and the results were compared with the dry modes of the structure at a fuel level of 0 mm. The simulation results of frequency and modal displacement are shown in Figure 13a,b.

Vibration theory states that the frequency of a structure mode is related to its mass, with a higher mass leading to a lower frequency. As shown in Figure 13a, with the increase in filled fuel level, the modal frequency of the structure remarkably decreased, which was due to the attached mass *M_A_* of the fuel. Figure 13b shows that, with the increase in filled fuel level, the modal displacement of the same specimen gradually decreased, and the modal displacement in the fuel medium was significantly larger than that in the air medium. The larger the modal displacement of the specimen, the larger is the stress amplitude and, hence, the specimen life in the fuel medium would be shorter than that in the air medium, which was consistent with the test results. The large modal displacement of the specimen in the fuel medium was due to the attached mass *M_A_* of the fuel. At the same acceleration, a specimen in the fuel medium would be subjected to a larger force owing to the attached mass *M_A_* than in the air medium; hence, the specimen in the fuel medium had a larger vibration amplitude with a shorter life.

### 5.2. Comparison of Fatigue Life Calculation Methods

The support in the test was rigid, and the 12 specimens did not affect each other when tested simultaneously. The stiffness value of the support material in the finite element model is very large, and the mass values of the specimens are small. Therefore, when a specimen failed, the vibration mode of the overall structure hardly changed. Based on the test and finite element simulation results, the specimen life was calculated using four approaches: narrowband approximation, simulated stress analysis, strain gauge method, and the analysis of measured average stress amplitude, and the calculation results were compared with the test results.

(1) Narrowband approximation

The probability density function *P*(*S_a_*) of the stress amplitude under sinusoidal excitation is equal to the probability density function *P*(*S_p_*) of the peak values, both obeying the Rayleigh distribution; hence, sinusoidal excitation generates a narrowband distribution. Narrowband distribution can be expressed by Equation (16) [30]:

(16) $$P\left(S_{a}\right)=P\left(S_{p}\right)=\frac{S_{a}}{\sigma_{RMS}^{2}}e^{-\frac{1}{2}{\left(\frac{S_{a}}{\sigma_{RMS}}\right)}^{2}}$$

where $\sigma_{RMS}$ is the root mean square of the stress process.

First of all, the harmonic response of the specimen fluid–solid coupling finite element model was calculated using a frequency range of 5–50 Hz. Then the acceleration-time course measured at the root of the air-medium specimen at an input acceleration of 8.55 g was converted to acceleration power spectral densities (PSDs). The harmonic response result was multiplied by the acceleration spectral density to obtain the stress spectral density. Then the stress spectral density function was calculated according to the narrow band distribution expression of the sinusoidal excitation. Finally, based on the fatigue life of the specimens obtained through Miner’s linear fatigue damage cumulation rule, the specimen life N_1_ are as shown in Table 9.

(2) Simulated stress analysis

The virtual mass method was employed to establish fluid–solid coupling models of the specimens for finite element simulation, which revealed the maximum stress at the specimen notch. Subsequently, the predicted life N_2_ of the specimens was obtained using the S–N curves of Q235-grade notched specimens [25,26], as shown in Table 10. 

(3) Strain gauge method

In the vibration fatigue tests, the strain of each specimen was measured using the strain gauge. The simulated stress at the specimen notch was approximately 9.5 times the stress at the gauge position; hence, the strain-time course at the gauge position was corrected for baseline drift and then multiplied by 9.5 to obtain the strain-time course at the specimen notch. Subsequently, the rainflow cycle counting method and linear damage theory were used to obtain the specimen life N_3_, as shown in Table 11.

(4) Analysis of measured average stress amplitude 

For the measured strain-time course, the average of the peak and trough values was calculated and then the average stress amplitude at the specimen notch was obtained. The predicted lifetime N_4_ of the specimens was obtained from the S–N curves of Q235-grade notch specimens by average stress amplitudes [25,26]. The S–N curves contained the elastic part and the plastic part, which took into account the effects of elasticity and plasticity, respectively. The average stress amplitude and the predicted lifetime N_4_ are shown in Table 12.

As shown in Table 9 and Table 10, the predicted lives N_1_ and N_2_ had the same relative deviation with respect to the test life, and the relative deviation of the life of most of the fuel-medium specimens was small. As shown in Table 11 and Table 12, the predicted life N_3_ of the air-medium specimens had smaller relative deviation with respect to the test life than the predicted life N_4_ did.

**Figure 14:**
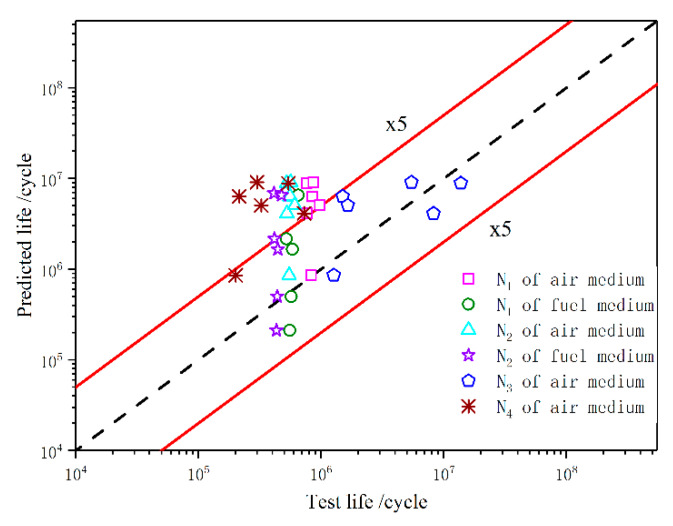
Real life and predicted life of the specimens.

In the double-logarithmic coordinate system, the predicted life was used as the abscissa and the test life was used as the ordinate, which generated relationship plots of real life versus predicted life for all the specimens, as shown in Figure 14 (the red solid lines represent the five-fold life range, the dashed line represents that the predicted life is equal to the real life). 

Generally, the prediction performance of a life calculation method is evaluated by the threshold ratio of 5 between the predicted life and test life, that is, if the data points of specimen life fall within the five-fold life range, a strong correlation is present [31]. As shown in Figure 14, all the predicted lives N_3_ fell within the five-fold life range, indicating that N_3_ was close to the true life. Most of the N_4_ data points were outside the five-fold life range, indicative of a large difference between the predicted life and real life. The predicted lives N_1_ and N_2_ of the fuel-medium specimens were mostly in the five-fold life range, indicating that, from the perspective of engineering practicality, it is possible to use narrowband approximation and simulated stress analysis to predict component fatigue life in a fuel medium.

## 6. Conclusions

In order to research the quantitative relationship between the fuel media and fatigue performance of the fuel tank material, the fluid-solid coupling finite element model was established based on the virtual mass method. The fatigue performance of the fuel tank, the fatigue life calculation methods and the influence of fuel on notch were studied through the fluid-solid coupling test under multi-stage loading and finite element simulation of the fuel tank base material. Based on the results obtained, it can be concluded that:

1. Under multi-stage loading, the life of Q235BF specimens in the air medium was significantly longer than that in the fuel medium, approximately 10 times as high as that in the fuel medium.
2. The specimens in pure bending tests were subject to the superposition of bending stress and compressive stress of the fuel at the crack tip. Compared with the air medium, the compressive stress of the fuel medium in the process of crack tip opening and closing led to higher crack propagation rates and fewer fatigue striations with severe fracture wear; the dimples in the instantaneous fracture zone were smaller and shallower, with higher irregularity in distribution, which provides reference for the calculation of the stress intensity factor at the crack tip of Q235BF specimens in the fuel media.
3. The energy spectrum analysis results of the crack source zone of the specimens in the air and fuel media indicated that this zone was not subject to fuel corrosion. In a short time the formation and propagation of cracks did not result from fuel erosion but resulted from the superposition of the bending stress at the crack tip with the compressive stress of the fuel.
4. The modal analysis indicates that the modal frequency and displacement of the structure gradually decreased with the increase in filled fuel level due to the influence of the attached mass *M*_A_. And the modal displacement of the fuel-medium specimens was larger than that of the air-medium specimens, which proves that the stress of the specimens in the fuel medium is larger than that in the air medium. In this condition, they have shorter life.
5. Comparing the four fluid–solid coupling model calculation methods, the predicted life derived from the measured strain of the air-medium specimens was close to the test life. Narrowband approximation and simulated stress analysis could well predict specimen life in the fuel medium which provides some reference for predicting the fatigue life of the fluid-solid model of the fuel tank.

Due to the limitation of experimental conditions, the stress intensity factor and crack propagation rate at the crack tip of the specimens in the fuel media have not been studied in depth. In addition, further research should be conducted to establish a crack propagation model by the crack propagation law to predict fatigue life.

## Figures and Tables

**Table 1:** Parameters of the MFLUID and ELIST control cards.

Card	Parameter			
MFLUID card	SID	CID	ZFS	RHO
	5	1	247.5	8.5 × 10^−10^
	ELIST1	ELIST2	PLANE1	PLANE2
	11	22	n	n
ELIST card	LID	E1	E2	E3
	11	29,356	THRU	35,799
	LID	E1	E2	E3
	22	35,800	THRU	37,910

**Table 2:** Small-scale model test results and modal simulation results.

Fuel Height	First-Order Frequency/Hz		Relative Deviation
	Frequency Sweep Test	Virtual Mass Method	
0	43	44.81	−4.21%
1/4	40	38.55	3.63%
1/2	37	34.88	5.73%
3/4	32	31.88	0.38%
1	29	30.24	−4.28%

**Table 3:** The chemical composition and physical and mechanical properties of Q235BF specimens.

Chemical Composition	C	Si	Mn	S	P	Cr	Ni	Cu
	≤0.2%	≤0.35%	≤1.4%	≤0.045%	≤0.045%	≤0.3%	≤0.3%	≤0.3%
Physical and mechanical properties	Density	Poisson’s ratio	Yield strength	Tensile strength	Elastic modulus	Form	Condition	Surface condition
	7850 kg/m^3^	0.25~0.33	235 MPa	370~50 MPa	200~210 GPa	Thin plate	Cold-rolled	Polished

**Table 4:** Simulation results of structural mode and static strength.

Order	Frequency/Hz	Input Acceleration	The First Principal Stress of Specimen/MPa
1	29.22	2.50 g	41.35
2	42.76	5.18 g	85.68
3	51.29	8.55 g	141.40
4	54.79	10.88 g	179.90
5	55.68	13.45 g	222.50

**Table 5:** Test results at an acceleration of 5.18 g.

Specimen in Air			Specimen in Fuel		
Serial No.	Number of Cycles	Fractured or Not	Serial No.	Number of Cycles	Fractured or Not
U_1	504,000	no	D_1	504,000	no
U_2	504,000	no	D_2	504,000	no
U_3	504,000	no	D_3	504,000	no
U_4	504,000	no	D_4	504,000	no
U_5	504,000	no	D_5	504,000	no
U_6	504,000	no	D_6	504,000	no

**Table 6:** Test results at an acceleration of 8.55 g.

Specimen in Air			Specimen in Fuel		
Serial No.	Number of Cycles	Fractured or Not	Serial No.	Number of Cycles	Fractured or Not
U_1	828,000	yes	D_1	180,000	yes
U_2	4,089,600	yes	D_2	2,131,200	yes
U_3	5,203,440	no	D_3	5,203,440	no
U_4	5,203,440	no	D_4	1,627,200	yes
U_5	5,203,440	no	D_5	468,000	yes
U_6	5,025,600	yes	D_6	5,203,440	no

**Table 7:** Test results at an acceleration of 13.45 g.

Specimen in Air			Specimen in Fuel		
Serial No.	Number of Cycles	Fractured or Not	Serial No.	Number of Cycles	Fractured or Not
U_1	/	yes	D_1	/	yes
U_2	/	yes	D_2	/	yes
U_3	196,560	yes	D_3	88,560	yes
U_4	208,080	yes	D_4	/	yes
U_5	59,760	yes	D_5	/	yes
U_6	/	yes	D_6	70,560	yes

**Table 8:** Life at an acceleration of 8.55 g converted from the test life at accelerations of 5.18 g and 13.45 g.

Specimen in Air					Specimen in Fuel					Ratio of Converted Life in the Air Medium to Converted Life in the Fuel Medium
Serial No.	Total Number of Cycles at a Certain Level of Acceleration			Number of Cycles When Converted to an Acceleration of 8.55 g	Serial No.	Total Number of Cycles at a Certain Level of Acceleration			Number of Cycles When Converted to an Acceleration of 8.55 g	
	5.18 g	8.55 g	13.45 g			5.18 g	8.55 g	13.45 g		
U_1	504,000	828,000	0	828,274	D_1	504,000	180,000	0	180,274	4.59
U_2	504,000	4,089,600	0	4,089,874	D_2	504,000	2,131,200	0	2,131,474	1.92
U_3	504,000	5,203,440	196,560	26,393,625	D_3	504,000	5,203,440	88,560	14,750,817	1.79
U_4	504,000	5,203,440	208,080	27,635,525	D_4	504,000	1,627,200	0	1,627,474	16.98
U_5	504,000	5,203,440	59760	11,646,068	D_5	504,000	468,000	0	468,274	24.87
U_6	504,000	5,025,600	0	5,025,874	D_6	504,000	5,203,440	70,560	12,810,349	0.39
Mean ratio of converted life in the air medium to converted life in the fuel medium										8.42

**Table 9:** Predicted life N_1_ of specimens under narrowband distribution.

Air-Medium Specimen				Fuel-Medium Specimen			
Serial No.	Predicted Life	Test Life	Relative Deviation/%	Serial No.	Predicted Life	Test Life	Relative Deviation/%
U_1	548,396	859,137	36.17	D_1	432,807	211,137	−104.99
U_2	526,732	4,120,737	87.22	D_2	415,714	2,162,337	80.77
U_3	523,560	8,877,482	94.10	D_3	412,797	6,875,886	94.00
U_4	564,493	9,090,986	93.79	D_4	444,346	1,658,337	73.21
U_5	558,347	6,342,127	91.20	D_5	438,596	499,137	12.13
U_6	608,273	5,056,737	87.97	D_6	476,077	654,2287	92.72

**Table 10:** Predicted life N_2_ derived from simulated stress analysis.

Air-Medium Specimen.					Fuel-Medium Specimen				
Serial No.	Maximum Notch Stress/MPa	Predicted Life	Test Life	Relative Deviation/%	Serial No.	Maximum Notch Stress/MPa	Predicted Life	Test Life	Relative Deviation/%
U_1	186.89	828,700	859,137	3.54	D_1	202.47	555,313	211,137	−163.01
U_2	189.64	770,353	4,120,737	81.31	D_2	205.14	520,078	2,162,337	75.95
U_3	190.02	762,701	8,877,482	91.41	D_3	205.47	515,928	6,875,886	92.50
U_4	185.15	868,425	9,090,986	90.45	D_4	200.51	583,062	1,658,337	64.84
U_5	186.00	848,785	6,342,127	86.62	D_5	201.38	570,533	499,137	−14.30
U_6	180.80	978,071	5,056,737	80.66	D_6	196.11	651,507	6,542,287	90.04

**Table 11:** Predicted life N_3_ derived from measured strain.

Serial No.	U_1	U_2	U_3	U_4	U_5	U_6
Predicted life	1,267,925	8,233,555	13,766,084	5,456,433	1,497,578	1,649,322
Test life	859,137	4,120,737	8,877,482	9,090,986	6,342,127	5,056,737
Relative deviation/%	−47.58	−99.81	−55.07	39.98	76.39	67.38

**Table 12:** Measured average stress amplitude and predicted life N_4._

Serial No.	U_1	U_2	U_3	U_4	U_5	U_6
Average stress amplitude	303.63	191.83	203.58	228.99	244.63	225.25
Predicted life	201,015	727,411	540,365	300,110	215,682	325,866
Test life	859,137	4,120,737	8,877,482	9,090,986	6,342,127	5,056,737
Relative deviation/%	76.60	82.35	93.91	96.70	96.60	93.56
